# Supplementary material for: Influence of smartphone addiction on sleep quality of college students: The regulatory effect of physical exercise behavior
Source: PLoS One. 2024 Jul 26;19(7):e0307162. doi: 10.1371/journal.pone.0307162 (PMC11280214; doi:10.1371/journal.pone.0307162)
Supplement: Attachment — Submitted filename: Response to Reviewers.docx [file pone.0307162.s002.docx]

Draft（PONE-D-23-21239）

Influence of Smartphone Addiction on Sleep Quality of College Students: The Regulatory Effect of Physical Exercise Behavior

24-Jul-2023

Dear Dr. li,

Thank you for submitting your manuscript to PLOS ONE. After careful consideration, we feel that it has merit but does not fully meet PLOS ONE’s publication criteria as it currently stands. Therefore, we invite you to submit a revised version of the manuscript that addresses the points raised during the review process.

Please submit your revised manuscript by Sep 28 2023 11:59PM. If you will need more time than this to complete your revisions, please reply to this message or contact the journal office at plosone@plos.org. When you're ready to submit your revision, log on to https://www.editorialmanager.com/pone/ and select the 'Submissions Needing Revision' folder to locate your manuscript file.

Please include the following items when submitting your revised manuscript:

A rebuttal letter that responds to each point raised by the academic editor and reviewer(s). You should upload this letter as a separate file labeled 'Response to Reviewers'.

A marked-up copy of your manuscript that highlights changes made to the original version. You should upload this as a separate file labeled 'Revised Manuscript with Track Changes'.

An unmarked version of your revised paper without tracked changes. You should upload this as a separate file labeled 'Manuscript'.

If you would like to make changes to your financial disclosure, please include your updated statement in your cover letter. Guidelines for resubmitting your figure files are available below the reviewer comments at the end of this letter.

If applicable, we recommend that you deposit your laboratory protocols in protocols.io to enhance the reproducibility of your results. Protocols.io assigns your protocol its own identifier (DOI) so that it can be cited independently in the future.

For instructions see:https://journals.plos.org/plosone/s/submission-guidelines#loc-

laboratory-protocols.

Additionally, PLOS ONE offers an option for publishing peer-reviewed Lab Protocol articles, which describe protocols hosted on protocols.io. Read more information on sharing protocolsat https://plos.org/protocols?utm_medium=editorial-email&utm_

source=authorletters&utm_campaign=protocols.We look forward to receiving your revised manuscript.

Kind regards,

Md. Saiful Islam, BPH, MPH

Academic Editor

PLOS ONE

College of Sports Science, Nantong University

Nantong，226019, China

Aug.15th,2023

Journal: PLOS One

Article type: Original Research

Manuscript ID: Draft（PONE-D-23-21239）

Manuscript title: Influence of Smartphone Addiction on Sleep Quality of College Students: The Regulatory Effect of Physical Exercise Behavior

Dear Editors and Reviewers:

Thank you for your letter and the reviewer’s comments regarding our manuscript, “Influence of Smartphone Addiction on Sleep Quality of College Students: The Regulatory Effect of Physical Exercise Behavior”(PONE-D-23-21239).These comments have been invaluable and helpful in revising and improving our paper, as well as important guidance for our research. We have carefully studied these comments and made revisions, which we hope will be recognized. The revised parts are highlighted in red in the paper. The corrections in the paper and the responses to the reviewer’s comments are as flowing:

Reviewer #1:

1.Your English writing needs further improvement. The language in submitted articles must be clear, correct, and unambiguous.

Response:We recognize your concern for the clarity and correctness of our English writing. We apologize for any ambiguities or errors that may have occurred. As a result, we have sought the assistance of a professional academic editing agency to ensure the accuracy and fluency of the language in this revision. We will be working closely with them to meticulously proofread and revise the paper.Thank you for your suggestion, and we will strive to ensure the language quality of the paper, as evidenced by the proof of editing provided below.

2.Your paper layout needs further beautification. For example, formula (1) is too large for the paper.

Response:We appreciate your feedback on the layout of our paper. We understand that the font size of Equation (1) is too large, making the article aesthetically unappealing. We will carefully adjust the font size and formatting to ensure that all formulas and charts are visually appealing while maintaining readability. Thank you for your suggestion, and we will strive to make improvements accordingly.

Reviewer #2：

1. This is a cross-sectional study, and the study design does not allow for reference to causal relationships.

Response:Thank you for your review and valuable feedback on our research titled "Influence of Smartphone Addiction on Sleep Quality of College Students: The Regulatory Effect of Physical Exercise Behavior" We greatly appreciate your comments and suggestions regarding the quality of research and data analysis.

We only discussed potential mechanisms of influence and did not include a discussion of causality in this research program. Additionally, we provided an explanation of the limitations of our study in the discussion section.

Thank you for your valuable feedback once again.

2. The study participants were students affiliated with a single university, making the study less representative.

Response:Thank you very much for your question about the representativeness of the sample. Although the participants in this study all come from the same university, they represent different majors and come from various provinces, cities and counties across China. Nonetheless, we recognize that the sample still has certain limitations and cannot fully represent the entire Chinese college student population.. For future studies, we will consider expanding the sample size and covering more schools and districts to improve the representativeness of the study. Thank you for your reminder and feedback.

3.The analysis only identified simple correlations and did not adjust for several potential confounders.

Response:Thank you very much for your question. This study only addresses the mechanisms of the moderating effects of physical exercise behavior and does not include explanations regarding confounding factors. Thank you for your question.

Participants
1. The authors should provide an overview of Nantong University so that readers can assess representativeness.

Response:Thank you very much for your feedback. We have included line numbers 103-106 in the article to provide additional information on the location and background of Nantong University, as well as when it was founded and its position in the education system in order to provide readers with a better understanding of the basics of Nantong University. Thank you for your suggestion.
2. The authors should describe the study participants in detail, including in the text and a table.

Response:Thank you very much for your question. We have added Table 1, which presents the sample distribution, and detailed textual explanations in lines 110-113 of the article. so that readers can have a more detailed understanding of the basic information of the study participants. We appreciate your suggestion and have made the necessary modifications.
3. The authors should provide an in-depth discussion of one of the major limitations of this study, representativeness, in the discussion section.

Response:Thank you very much for your question. We will discuss the representativeness of the sample in the discussion section. This can be found at lines 428-431 of the article.
4. I did not understand the description of the sample size calculation method.

Response:Thank you very much for your question about the sample size calculation method. The calculation method used in this article is based on the approach mentioned in the literature by Chinese scholar Shao Zhiqiang. In this method, α corresponds to the formula zα, and α is generally set to 0.05. For a two-tailed test, z0.05 is 1.96, and for a one-tailed test, z0.05 is 1.64. δ represents the allowable error, which is usually determined based on experience and should not be too large. p represents the prevalence, which is the overall population prevalence and is typically set at 0.05. The finite population size N depends on the target population, such as the total number of students in a school or patients in a hospital.

Measurement Methods
5. The authors should provide a table of data on study participant characteristics, such as obesity rate, smoking and alcohol consumption rates, and potential confounders that may influence the relationship between smartphone addiction and sleep, such as academic achievement, financial ability, commuting time to university, and living environment, if they measured these factors.

Response:Thank you very much for your question. As the questionnaire did not collect data on factors such as obesity rate, smoking and drinking rates, academic achievement and financial ability , this will be noted in the limitations of this study.
6. If the authors did not have access to the characteristics and potential confounders of the study participants, this should be noted in the discussion section as a limitation of the study.

Response:Thank you very much for your question. Since this article did not collect detailed data on features and confounding factors during data collection, we will point this out in the limitations of the discussion section,lines413-417.
7. The authors should cite the original article on the physical activity assessment study.

Response:Thank you very much for your question. We will add the original reference from Japanese scholar Hashimoto Kimio to the section on the sports activity level scale in section 2.2.3.
Statistical Analysis
8. The authors should adjust for confounders using multivariable analysis methods.

Response: Thank you very much for your question. This study only focuses on the regulatory mechanisms of physical exercise and does not involve explanations regarding confounding factors. Thank you for your question.

Reviewer #3: In this study, the authors investigate the influence of smartphone addiction on the sleep quality of college students: the regulatory effect of physical exercise behavior. The study draws attention with its sample size. My concerns about the study are listed below.

1.As the authors know, there are many studies conducted in this area (33. Sahin S, Ozdemir K, Unsal A, Temiz N. Evaluation of mobile phone addiction level and sleep quality in university students. Pak J Med Sci. 2013;29(4):913-8. doi: 10.12669/pjms.294.3686. PubMed PMID: WOS:000322619000005.)

More up-to-date resources in WOS could have been used (Buke, M., Egesoy, H., & Unver, F. (2021). The effect of smartphone addiction on physical activity level in sports science undergraduates. Journal of bodywork and movement therapies, 28, 530-534).

Response: Thank you very much for your guidance. We have replaced reference 33 in this study.
2.The concept of hypothesis is misused. H1-H2-H3 were questions that the authors sought answers to.

Response: Thank you very much for your question. This study will exclude the hypothesis section and only focus on seeking answers to the questions proposed by H1-H2-H3.

3. Mobile Phone Addiction Tendency Scale (MPATS) and Physical Activity 151 Rating Scale-3 (PARS-3) should be included in the summary.

Response: Thank you very much for your question. We will add the Mobile Addiction Tendency Scale and the Physical Activity Level Scale in the abstract, specifically at line number 31-33.
4.Spelling and punctuation in the text should be checked. Use the dot after the closed parenthesis.

Response: Thank you very much for your question. We have carefully checked the spelling and punctuation in the manuscript again. We have also sought assistance of a professional proofreading agency to ensure the coherence and correctness of the language in the article.
