# Supplementary material for: Influence of smartphone addiction on sleep quality of college students: The regulatory effect of physical exercise behavior
Source: PLoS One. 2024 Jul 26;19(7):e0307162. doi: 10.1371/journal.pone.0307162 (PMC11280214; doi:10.1371/journal.pone.0307162)
Supplement: Attachment — Submitted filename: Response to Reviewer.docx [file pone.0307162.s003.docx]

PONE-D-23-21239R1

Influence of Smartphone Addiction on Sleep Quality of College Students: The Regulatory Effect of Physical Exercise Behavior

PLOS ONE

Dear Dr. li,

Thank you for submitting your manuscript to PLOS ONE. After careful consideration, we feel that it has merit but does not fully meet PLOS ONE’s publication criteria as it currently stands. Therefore, we invite you to submit a revised version of the manuscript that addresses the points raised during the review process.

I saw the comments of the reviewers, both in the previous round of review and in the present one. Some comments are somewhat conflicting, but I believe, based on their judgments, that there are minor changes that could be made to improve the work. I therefore invite you to further revise the paper, which can improve a lot if you follow these suggestions.

Please submit your revised manuscript by Mar 22 2024 11:59PM. If you will need more time than this to complete your revisions, please reply to this message or contact the journal office at [plosone@plos.org](mailto:plosone@plos.org). When you're ready to submit your revision, log on to https://www.editorialmanager.com/pone/ and select the 'Submissions Needing Revision' folder to locate your manuscript file.

Please include the following items when submitting your revised manuscript:

A rebuttal letter that responds to each point raised by the academic editor and reviewer(s). You should upload this letter as a separate file labeled 'Response to Reviewers'.

A marked-up copy of your manuscript that highlights changes made to the original version. You should upload this as a separate file labeled 'Revised Manuscript with Track Changes'.

An unmarked version of your revised paper without tracked changes. You should upload this as a separate file labeled 'Manuscript'.

If you would like to make changes to your financial disclosure, please include your updated statement in your cover letter. Guidelines for resubmitting your figure files are available below the reviewer comments at the end of this letter.

If applicable, we recommend that you deposit your laboratory protocols in protocols.io to enhance the reproducibility of your results. Protocols.io assigns your protocol its own identifier (DOI) so that it can be cited independently in the future. For instructions see: [https://journals.plos.org/plosone/s/submission-guidelines#loc-laboratory-protocols](https://journals.plos.org/plosone/s/submission-guidelines#loc-laboratory-protocols). Additionally, PLOS ONE offers an option for publishing peer-reviewed Lab Protocol articles, which describe protocols hosted on protocols.io. Read more information on sharing protocols at <https://plos.org/protocols?utm_medium=editorial-email&utm_source=authorletters&utm_campaign=protocols>.

We look forward to receiving your revised manuscript.

Kind regards,

Giulia Ballarotto
Academic Editor
PLOS ONE

College of Sports Science, Nantong University

Nantong，226019, China

Feb.8th,2024

Journal: PLOS One

Article type: Original Research

Manuscript ID: Draft（PONE-D-23-21239）

Manuscript title: Influence of Smartphone Addiction on Sleep Quality of College Students: The Regulatory Effect of Physical Exercise Behavior

Dear Editors and Reviewers:

Thank you for your letter and the reviewer’s comments regarding our manuscript, “Influence of Smartphone Addiction on Sleep Quality of College Students: The Regulatory Effect of Physical Exercise Behavior”(PONE-D-23-21239).These comments have been invaluable and helpful in revising and improving our paper, as well as important guidance for our research. We have carefully studied these comments and made revisions, which we hope will be recognized. The revised parts are highlighted in red in the paper. The corrections in the paper and the responses to the reviewer’s comments are as flowing:

Reviewer #4: Abstract:
"explore its regulating effect by introducing physical exercise" This refer as if the study an interventional one. Please rephrase in a way that make it a cross sectional study.

Response: Thank you for your insightful comments and for emphasizing the need for clarity regarding our study design. As you have correctly pointed out, the original wording suggested an interventional approach, which was not in line with the intent of our study.

I have revised the language in the manuscript to more accurately convey that our research is a cross-sectional analysis. We have rephrased the purpose of our study to underscore the examination of associations rather than implying any intervention. Moreover, I have highlighted the correlation between physical exercise and the relationship between smartphone addiction and sleep quality to further clarify that our study design is observational in nature.

I hope these modifications address your concerns and faithfully communicate the cross-sectional nature of our study. Please feel free to make any further suggestions if necessary. For these changes, please refer to lines 26-30.

"r=-0.101", here do you mean r-squared of the regression? if not, please report r-squared instead of r value.

Response: Thank you for your inquiry. The "r=-0.101" mentioned here refers to the correlation coefficient result, not the r-squared value of the regression. You can find the report on r-squared in lines 37 to 39 of the revised manuscript. Should you have any further questions or require additional information, please feel free to let me know.

Introduction:
-"problems to people.[1]" please adjust the reference style.

Thank you for bringing this to my attention. I have corrected the reference style as per your suggestion. The updated reference can now be found in the revised manuscript at line number 49.

- "generates negative emotions, such as fatigue and procrastination." where is the reference?

Response:Thank you for your question and for pointing out the oversight. We have added the appropriate reference for this statement. Please refer to line 67 of the revised manuscript for the specific correction and the added citation. If there are any other concerns or clarifications needed, do not hesitate to contact us.

- "The current study on smartphone addiction mainly focus on mental health and
hygiene", Do you mean your study? Do you mean mental hygiene? Clarify please.

Response:Thank you for seeking clarification. When I referred to "The current study on smartphone addiction mainly focus on mental health and hygiene," I was intending to summarize the general trend of research in the area of smartphone addiction. Upon further examination, we recognized that there is insufficient evidence in the literature to substantiate the focus on mental health and hygiene specifically in relation to smartphone addiction. Consequently, we have opted to eliminate this statement from our manuscript to ensure accuracy. This change can be found in the revised version, where lines 67-69 have been removed.

- Please include studies that previously assessed the relationship between smartphone addiction and sleep quality (such as https://www.ncbi.nlm.nih.gov/pmc/articles/PMC9879389/), and those also assessed physical activity and its effect on sleep quality? Is there any study in China? Please highlight in the introduction to clarify the gap in knowledge.

Response: Thank you for your insightful comments and your suggestion to include prior studies that assessed the relationship between smartphone addiction and sleep quality, as well as the intersection of physical activity and its effect on sleep quality.

In response to your request, I will enrich the introduction section by discussing several pivotal studies that have investigated these important themes. For example, I will reference the study you provided (https://www.ncbi.nlm.nih.gov/pmc/articles/PMC9879389/), which offers a comprehensive analysis of how excessive smartphone use may detrimentally affect sleep quality.

Furthermore, I will make an effort to highlight research conducted in China regarding this topic. Indeed, scholars in China have undertaken numerous studies examining the effects of smartphone addiction on various aspects of health and daily life, including sleep quality. I will identify and include relevant Chinese studies to underline the existing research in the area, which will help to illustrate the current gap in knowledge that our study aims to address.

By integrating these studies, the introduction will clearly state the contribution of our research within the broader context of existing literature. This will not only fortify the backdrop against which our study stands but will also elucidate the necessity for further research to bridge the knowledge gap identified.For the relevant revisions, please refer to lines 86-92.

- "What are the characteristics of contemporary college students' smartphone addiction, sleep quality, and physical exercise?" This is not the correct way to write a scientific hypothesis. In fact, the all the of them need to be rewritten or to change it into study objective.

Response: Thank you for your keen observation regarding the construction of our research questions and hypotheses. Upon reflection, we concede that the initial formulation of our hypotheses more closely resembled questions, which could lead to misinterpretation and are not in line with the traditional scientific format.

In light of your comment, we have revised our study objectives to clearly delineate our aims and dispel any confusion. The hypotheses have been reformulated into objective statements that clearly chart the course of our investigation and lay the groundwork for operationalization and empirical testing. We believe these adjustments will elucidate the intent and scope of our research, ensuring that our objectives are more precise and methodologically robust.

We are grateful for the opportunity to integrate your invaluable feedback, which has undoubtedly enriched the framework of our research. We trust that these revisions will meet with your approval and will elevate the quality of our study. The pertinent revisions can be referenced on lines 107-112.

-remove the word contemporary and rephrase the sentence.

Response: Thank you for your suggestion. We have removed the word "contemporary" and rephrased the sentence to better reflect our intended meaning. The revised sentence maintains the essence of the original statement while ensuring clarity and conciseness. We appreciate your attention to detail and agree that this modification improves the manuscript. Please see the revised sentence on line 109 of the updated document.

Methods:

- "The scales used in the" do you mean questionnaire? replace.

Response: Thank you for your comment. Indeed, when we referred to "the used scales," we were specifically alluding to the scales within the questionnaire. We have made the necessary correction in the text to express this unambiguously. The term "scales" previously mentioned in the text has now been replaced with "questionnaire." Please refer to line 122 in the revised manuscript for this correction. We appreciate your meticulous review and valuable feedback.

- "effective rate of 78%." change it into response rate.

Response: Thank you for your attentive reading of our manuscript and the suggestion to clarify our terminology. We agree that the term “effective rate of 78%” could be misunderstood and that “response rate” is more appropriate and commonly utilized in this context.

We have updated the manuscript to reflect this change. The phrase “effective rate of 78%” has now been revised to “response rate of 78%” to ensure clarity and align with standard research terminology.

Please refer to the updated line 131 in the revised manuscript where this correction has been applied.

We appreciate your valuable input and believe that this modification improves the accuracy and readability of our study.

- "Given that the total population is 48,209 people" the total population of all university students in China, or in that university or what? if you have the reference that will be great.

Response: Thank you very much for your question. The total population of 48,209 people mentioned here refers to the total number of enrolled students at Yangzhou University as of 2023, not the total number of university students in China. You can find the relevant information on the total number of students on the official website of Yangzhou University, which is http://www.yzu.edu.cn/xxgk/xxjj.htm. We apologize for not making this clear in our previous description in the article, and we have made the necessary revisions in the revised manuscript. Please refer to the updated line number 135.

Research Findings

- please include a table about demographic data of the participants.

Response: Thank you for your inquiry. I can confirm that the demographic data of the participants is indeed included in the article. We have organized this information into three sections: gender, academic level, and age. Please refer to line 128 for the relevant revisions.

- Also, I think you have to put a descriptive statistics to all the variables, in continuous and categorical format before going into analysis. This will answer the research hypothesis number 1 in your research.

Response: Thank you very much for your query. We have indeed classified the variables in the scale into their respective categories. For more detailed information, please refer to the revised manuscript at lines 157-158, 173-174, and 186. We believe this classification aligns with the requirements to adequately address research hypothesis number 1.

- Table 1 design is little confusing, I wish you can adjust it so it can be read easily. For example, you have in the row three sections total, gender and academic year, so please make that clear.

Response: Thank you for your meticulous review and valuable suggestions. We have made a series of improvements to Table 1 as per your guidance. Firstly, we have enhanced the distinction among the various categorization fields, ensuring that the “Total,” “Gender,” and “Grade Level” sections are clear and well-differentiated. Additionally, to further improve the table’s conciseness and readability, we have removed the “Age Group” information.

Please review the revised manuscript at your convenience. We highly value your feedback and hope that our modifications meet your approval.

We are sincerely grateful for your contribution to our work and look forward to your response.

- Table 2 is so confusing with the numbers, please either change the layout, or use 1 or 2 decimals maximum in the table. Remove F-value, as it is confusing.

Response: Thank you very much for your feedback. In reviewing Table 2, we noticed that it did not list the F-values; hence, we presume you might be referring to the issue with the F-values in Table 3. Following your suggestion, we have removed the F-values from Table 3 and adjusted the numeric values to two decimal places for increased clarity and conciseness.

We hope these changes address your concerns and enhance the readability of the data presentation. For details on the revisions, please refer to line number 225.

Thank you again for your valuable comments.

- In Table 4 please put footnote for what model 1 include, and what model 2 include.

Response: Thank you for inquiring about the contents of Table 4. As per your request, we will ensure the provision of appropriate footnotes detailing the components of Model 1 and Model 2 to enhance the clarity of our analysis for the reader's comprehension. We will make certain that these details are succinctly summarized in the footnotes to aid readers in better understanding the composition and outcomes of the models in our study. For specific modifications, please refer to lines 253-255.

- In Table 4 please clarify if you used the data in its continuous format or categorical one, because that was not clear to reader.

Response: Thank you very much for your inquiry regarding the nature of the data used in Table 4. To clarify, the data presented in Table 4 have been processed as categorical variables. We are extremely grateful for your attention to detail and understand the importance of clarifying this to our readers. To eliminate any ambiguity, we will revise the manuscript to explicitly state that the variables in Table 4 have been analyzed as categorical variables. We will make the necessary changes at lines 234-235 of the manuscript.

Discussion:

- "previous studies[16]." in what country?

Response: Thank you for your question regarding “previous studies[16].” I realize the importance of providing the geographic context of referenced studies for the reader’s understanding.

In response to your query, the previous studies mentioned are based in the United States. To clarify this in our manuscript, we have now included the country where the referenced research was conducted. You can find this update in lines 273-274 of the manuscript.

I appreciate your keen eye for detail and the opportunity to make our manuscript as informative and precise as possible.

-"Trevor Egli MS" do you mean that this is reference 16 please put the reference here.

Response: Thank you for your question, and I apologize for the oversight. I have now corrected the placement of the reference. Please see line 278 in the manuscript for the specific citation.

- In discussion from line 248-258, they were written without reference, if this is the author opinion, I think you should write, it is suggested or similar terms. This is applied to many parts in the discussion, where many sentences without reference. So I suggest that write what make it clear that this is your suggestion

Response: Thank you for your feedback. I understand your concerns regarding the lack of references for certain opinions expressed between lines 248-258 of the discussion section. I will take your suggestion and make it clear that these are our suggestions or personal viewpoints. I will address this issue throughout the relevant parts of the discussion to avoid confusion for the readers. For sentences without references, I will add phrases such as "we suggest" or "based on our observation" to clearly indicate that it reflects the authors' perspectives. Once again, I appreciate your valuable comments. For specific modifications, please refer to the manuscript lines 278-279 and 296-297.

- Your discussion is long, and I think you need to make subheading for it.

Response:Thank you for your constructive feedback on the discussion section of our manuscript. I appreciate your suggestion to incorporate subheadings to improve the readability and structure of our text.

I agree that the discussion section is rich in content, and adding subheadings will help readers better navigate and understand the various arguments and conclusions we have presented. We will revise this part by adding relevant subheadings that reflect the core points and themes of our discussion. Such restructuring will undoubtedly make the content more understandable and focused.

We will ensure that each subheading is short and descriptive, allowing readers to quickly grasp the main idea of the subsequent paragraphs. We believe these changes will enhance the overall clarity of the manuscript, and we thank you for pointing this out.

For specific modifications, please refer to lines 272-273, 319-320, 360-361, 398, and 411 of the manuscript.
