# Supplementary material for: Influence of smartphone addiction on sleep quality of college students: The regulatory effect of physical exercise behavior
Source: PLoS One. 2024 Jul 26;19(7):e0307162. doi: 10.1371/journal.pone.0307162 (PMC11280214; doi:10.1371/journal.pone.0307162)
Supplement: Attachment — Submitted filename: Response to Reviewers.docx [file pone.0307162.s004.docx]

College of Sports Science, Nantong University

Nantong，226019, China

May.10th,2024

Journal: PLOS One

Article type: Original Research

Manuscript ID: Draft（PONE-D-23-21239R2）

Manuscript title: Influence of Smartphone Addiction on Sleep Quality of College Students: The Regulatory Effect of Physical Exercise Behavior

Dear Editors and Reviewers:

Thank you for your letter and the reviewer’s comments regarding our manuscript, “Influence of Smartphone Addiction on Sleep Quality of College Students: The Regulatory Effect of Physical Exercise Behavior”(PONE-D-23-21239R2).These comments have been invaluable and helpful in revising and improving our paper, as well as important guidance for our research. We have carefully studied these comments and made revisions, which we hope will be recognized. The revised parts are highlighted in red in the paper. The corrections in the paper and the responses to the reviewer’s comments are as flowing:

Journal Requirements:

Please review your reference list to ensure that it is complete and correct. If you have cited papers that have been retracted, please include the rationale for doing so in the manuscript text, or remove these references and replace them with relevant current references. Any changes to the reference list should be mentioned in the rebuttal letter that accompanies your revised manuscript. If you need to cite a retracted article, indicate the article’s retracted status in the References list and also include a citation and full reference for the retraction notice.

Response: As per your request, we have thoroughly reviewed our reference list to ensure its completeness and accuracy. We are pleased to confirm that none of the papers we cited have been retracted. Therefore, there is no need for us to provide reasons for citing retracted papers or to replace any references.

Furthermore, to enhance the accuracy and accessibility of the cited works, we have updated the reference list by adding DOI numbers to references 23, 24, and 28. Thank you for your guidance in helping us maintain high standards in our scholarly reporting. Should you require further adjustments or need more clarification regarding our submission, please feel free to contact us.

Reviewer #3: This study examined the effect of smartphone addiction on college students' sleep quality: the moderating effect of physical exercise behavior. The corrections I requested have been made, thank you.

Response: Thank you for your feedback. I am pleased to confirm that the necessary corrections have been made. Should you have any further queries or require additional clarifications, please feel free to contact us.

Reviewer #5: Thank you very much for the possibility to review the study "Influence of Smartphone Addiction on Sleep Quality of College Students: The Regulatory Effect of Physical Exercise Behavior". The work is interesting and the revisions carried out have allowed an improvement of the work. I think it has improved significantly. At the same time, however, there are some aspects that should be improved. In particular, the authors focused on university students, highlighting the association between smartphone addiction and the quality of sleep. It would be useful to be able to discuss the results of the study (but also an in-depth analysis in the introductory part) of the evolutionary phase in which the participants of the study find themselves. In particular, there is a large literature on emerging adulthood that should be explored in order to better understand the results of the study. Some specific studies are recommended:

- Arnett, J. J. (2007). Emerging adulthood: What is it, and what is it good for?. Child development perspectives, 1(2), 68-73.

- Ballarotto, G., Marzilli, E., Cerniglia, L., Cimino, S., & Tambelli, R. (2021). How does psychological distress due to the COVID-19 pandemic impact on internet addiction and Instagram addiction in emerging adults?. International journal of environmental research and public health, 18(21), 11382.

- Hochberg, Z. E., & Konner, M. (2020). Emerging adulthood, a pre-adult life-history stage. Frontiers in endocrinology, 10, 918.

- Tambelli, R., Cimino, S., Marzilli, E., Ballarotto, G., & Cerniglia, L. (2021). Late adolescents’ attachment to parents and peers and psychological distress resulting from COVID-19. A study on the mediation role of alexithymia. International Journal of Environmental Research and Public Health, 18(20), 10649.

- Griffioen, N., Scholten, H., Lichtwarck-Aschoff, A., Van Rooij, M., & Granic, I. (2021). Everyone does it—differently: A window into emerging adults’ smartphone use. Humanities and Social Sciences Communications, 8(1), 1-11.

Response: we thank you for your detailed review and valuable suggestions on our research. Based on your feedback, we have conducted an in-depth analysis of the introduction section and integrated the literature you recommended to enhance our understanding and background of the study topic. These revisions not only deepened the theoretical depth of our paper but also provided a more solid theoretical foundation for our research findings. Once again, thank you for your careful guidance and suggestions, which have been critical in enhancing the quality and depth of our study. Please refer to lines 59-73, 325-330, 442-447, and 452-453 of the article for specific modifications.
